# Role of right temporoparietal junction for counterfactual evaluation of partner’s decision in ultimatum game

**DOI:** 10.1093/cercor/bhac252

**Published:** 2022-06-19

**Authors:** Akitoshi Ogawa, Saki Asano, Takahiro Osada, Masaki Tanaka, Reia Tochigi, Koji Kamagata, Shigeki Aoki, Seiki Konishi

**Affiliations:** Department of Neurophysiology, Juntendo University School of Medicine, 2-1-1 Hongo, Bunkyo-ku, Tokyo 113-8421, Japan; Department of Neurophysiology, Juntendo University School of Medicine, 2-1-1 Hongo, Bunkyo-ku, Tokyo 113-8421, Japan; Department of Neurophysiology, Juntendo University School of Medicine, 2-1-1 Hongo, Bunkyo-ku, Tokyo 113-8421, Japan; Department of Neurophysiology, Juntendo University School of Medicine, 2-1-1 Hongo, Bunkyo-ku, Tokyo 113-8421, Japan; Department of Neurophysiology, Juntendo University School of Medicine, 2-1-1 Hongo, Bunkyo-ku, Tokyo 113-8421, Japan; Department of Radiology, Juntendo University School of Medicine, 2-1-1 Hongo, Bunkyo-ku, Tokyo 113-8421, Japan; Department of Radiology, Juntendo University School of Medicine, 2-1-1 Hongo, Bunkyo-ku, Tokyo 113-8421, Japan; Department of Neurophysiology, Juntendo University School of Medicine, 2-1-1 Hongo, Bunkyo-ku, Tokyo 113-8421, Japan

**Keywords:** functional magnetic resonance imaging, human, functional connectivity, dynamic causal modeling, transcranial magnetic stimulation

## Abstract

Humans assess the distributions of resources based on their aversion to unfairness. If a partner distributes in an unfair manner even though the partner had a less unfair distribution option, a recipient will believe that the partner should have chosen the counterfactual option. In this study, we investigated the neural basis for fairness evaluation of actual and counterfactual options in the ultimatum game. In this task, a partner chose one distribution option out of two options, and a participant accepted or rejected the option. The behavioral results showed that the acceptance rate was influenced by counterfactual evaluation (CE), among others, as defined by the difference of monetary amount between the actual and counterfactual options. The functional magnetic resonance imaging results showed that CE was associated with the right ventral angular gyrus (vAG) that provided one of convergent inputs to the supramarginal gyrus related to decision utility, which reflects gross preferences for the distribution options. Furthermore, inhibitory repetitive transcranial magnetic stimulation administered to the right vAG reduced the behavioral component associated with CE. These results suggest that our acceptance/rejection of distribution options relies on multiple processes (monetary amount, disadvantageous inequity, and CE) and that the right vAG causally contributes to CE.

## Introduction

Humans have a strong preference for fair distributions of resources. People are not only self-interested but also other regarding, motivated by inequity aversion (Fehr and Schmidt 1999). Inequity aversion in monetary distribution is simplified typically in a situation like the ultimatum game (UG) (Güth et al. 1982). In the UG, a partner and a recipient share a certain amount of money. The partner offers only one distribution option to the recipient, who then decides whether to accept or reject the option. If the recipient rejects the distribution option, both partner and recipient receive nothing. Therefore, from an economic perspective, the optimal decision for the recipient participants in the UG is to accept any distribution options, except zero distribution. However, it has been well replicated that recipients are likely to reject the distribution option where the participant receives less than the partner, i.e. disadvantageous inequity (DI), and fairer distribution options are more likely accepted (Sanfey et al. 2003; Knoch et al. 2006; Van’t Wout et al. 2006; Fehr and Camerer 2007; Koenigs and Tranel 2007; Yamagishi et al. 2009; Güroǧlu et al. 2010, 2011, 2014; Chang and Sanfey 2013; Corradi-Dell’Acqua et al. 2013; Crockett et al. 2013; Gabay et al. 2014; Holper et al. 2018; Inaba et al. 2018; Speitel et al. 2019; Hu and Mai 2021). As has been previously explained, the rejection is carried out to punish the unfair partner and enforce a certain consideration of fairness (Fehr and Schmidt 1999; Koenigs and Tranel 2007). Thus, DI represents an essential component that drives the recipient to positive responses in decision-making in the UG.

Another key component related to fairness also matters during making decisions to accept/reject a distribution option in the UG. In a revised version of the UG, a third party provides two distribution options, and a partner chooses one out of the two options (Falk et al. 2003; Güroǧlu et al. 2010). If a partner chooses a less fair distribution, but the partner also had a fairer distribution, a recipient will think that the partner could have chosen the fairer distribution. The counterfactual evaluation (CE), i.e. the difference between actual and counterfactual options, influences the decision to accept/reject a distribution option (Falk et al. 2003; Sandbu 2007; Nicklin et al. 2011; Li et al. 2017). When the partner intends to get more money selfishly, the recipient is more likely to reject the option.

There are two main approaches to human economic behavior with respect to fairness (Sandbu 2007). One is called outcome fairness, which assumes that humans only care about the actual distribution, without reference to the process that produced the outcome distribution, i.e. involving DI but not CE (Fehr and Schmidt 1999; Bolton and Ockenfels 2000; Andreoni and Miller 2002). The other is called contextual fairness, which assumes that the same allocation can be judged differently, depending on the context that produced the distribution, i.e. involving DI and CE (Andreoni et al. 2002; Falk et al. 2003; Dufwenberg and Kirchsteiger 2004).

Previous neuroimaging studies have revealed that various brain regions are involved in rejecting a distribution option with DI in the UG (Sanfey et al. 2003; Rilling et al. 2004; Halko et al. 2009; Güroǧlu et al. 2010; Hollmann et al. 2011; Fliessbach et al. 2012; Harlé et al. 2012; Corradi-Dell’Acqua et al. 2013; Gabay et al. 2014; Haruno et al. 2014; Cheng et al. 2017; Hétu et al. 2017; Holper et al. 2018). Among these regions, the right temporoparietal junction (rTPJ) is known to play an essential role in making accept/reject decisions based on DI in the UG (Rilling et al. 2004; Halko et al. 2009; Güroǧlu et al. 2010). The rTPJ is involved in the processing of mentalization or “theory-of-mind,” that is, the ability to infer the others’ agency and mental states (Saxe and Kanwisher 2003; Saxe and Wexler 2005; Saxe 2006; Völlm et al. 2006; Van Overwalle 2009; Van Overwalle and Baetens 2009; Schnell et al. 2011; Van Overwalle 2011; Carter et al. 2012; Koster-Hale and Saxe 2013; Takahashi et al. 2014; Ogawa and Kameda 2019). The rTPJ is also associated with the decision-making of social distribution (Kameda et al. 2016) and that for others (Ogawa et al. 2018). A previous neuroimaging study suggested that the processing of counterfactual options is associated with the mentalizing network including the rTPJ (Van Hoeck et al. 2013). However, it remains elusive how CE is represented in the brain and is integrated with DI to make a decision in the context of the UG.

**Fig. 1:**
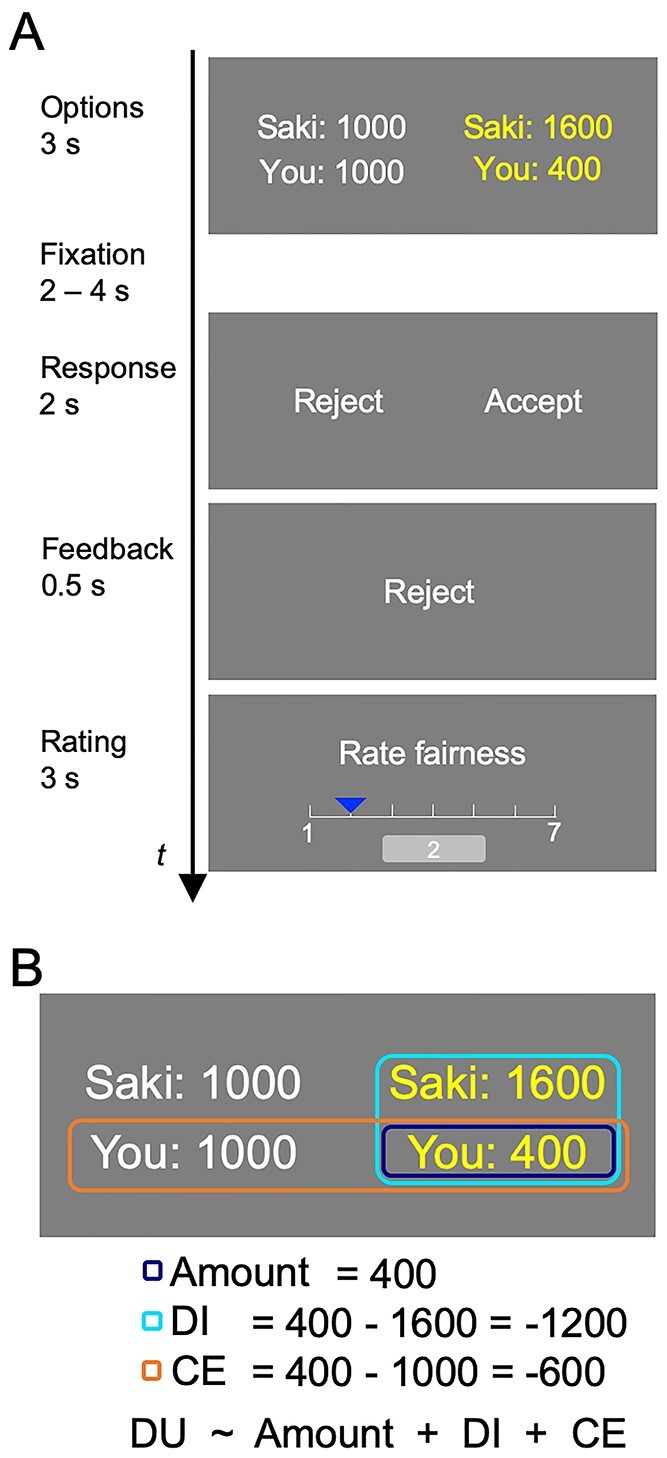
A modified version of the ultimatum game. A. Task trial. In each trial, two distribution options are presented for 3 s. one option, colored yellow, represents the actual distribution that the partner chose (actual option). The other option, colored white, represents the distribution that the partner did not choose (counterfactual option). The distribution options include the amounts of money for the partner (i.e. Saki) and the participant. Following the fixation period, participants are asked to accept or reject the actual option by pressing a button within 2 s. next, the participant’s response is presented for 0.5 s. the participants rate the current offer on 7-point scale. The inter-trial interval lasts 2, 3, or 4 s. B. Modeled components. The model of the participants’ decisions includes amount, disadvantageous inequity (DI), counterfactual evaluation (CE), and decision utility (DU).

In this study, we investigated CE of actual and counterfactual distribution options in the UG using functional magnetic resonance imaging (fMRI) and transcranial magnetic stimulation (TMS). A partner chose one of two distribution options provided by a third party to offer it to a recipient participant, after which the recipient decided to accept or reject the distribution option (Fig. 1A). We modeled CE, as well as the DI, as fairness consideration that constitutes decision utility (DU), which reflects gross preference for the distribution options (Fig. 1B). A parametric modulation analysis of fMRI may be more suitable for segregating these components to assess the neural correlates of each component than a simple contrast analysis of the rejection vs. acceptance. We probed the neural basis of CE using the model-based analysis and tested via connectivity analyses how CE was integrated with DI into DU (cf. Suzuki and O’Doherty 2020), thereby leading to the recipient’s decision to accept/reject in the UG. Thereafter, we impaired CE by administering inhibitory repetitive TMS to the brain region associated with CE to reveal its causal role using a totally different set of participants from the fMRI experiment.

## Materials and methods

### Participants

Twenty right-handed participants without neurological or psychiatric illness participated in the fMRI experiment (8 men and 12 women; 21.3 ± 1.2 years (mean ± standard deviation), ranging from 20 to 25 years of age). Another set of twenty right-handed participants without neurological or psychiatric illness participated in the TMS experiment (7 men and 13 women; 21.5 ± 2.5 years, ranging from 20 to 29 years). Written informed consent was obtained from all participants prior to the start of experiments. Research Ethics Committee, Faculty of Medicine, Juntendo University approved the experimental procedures.

### Behavioral procedures

The task was a modified UG (Güth et al. 1982; Falk et al. 2003; Li et al. 2017). In each session, immediately before the beginning of the task, the partner’s name (i.e. Saki in this study) and picture were presented. In each trial (Fig. 1A), two distribution options were presented on the right and left for 3 s. Each option reflected the amount of money for the partner and the participant. The option that the partner chose was colored yellow (actual option), while the other option was colored white (counterfactual option). After a jittered fixation duration (2, 3, or 4 s), the participant indicated whether to accept or reject the proposal by pressing a button. The assignment of acceptance/rejection to the left/right button was randomly determined in each trial to prevent motor-related contamination in the neuroimaging analyses of acceptance vs. rejection contrast. If the participant rejected the proposal, both the participant and the partner obtained nothing. If the participant did not press a button, the offer was considered to have been accepted but was not used for subsequent analyses. Next, the participants were asked to rate the fairness of the distribution according to a seven-point scale (1 = absolutely unfair, 7 = absolutely fair).

The amount of money for the participants was equal to or less than that for the partner. In such experimental settings, previous studies demonstrated that the decision to accept was based on the amount of money for the participant (Amount) and the DI (Fehr and Schmidt 1999: Sanfey et al. 2003). In this study, we considered CE to be a third component influencing the decision to accept. DI was defined as the amount of money for the participant minus that for the partner in the actual option, while CE was defined as the amount of money for the participant in the actual option minus that in the counterfactual option (Fig. 1B).

Since Amount, DI, and CE are highly related, it remains difficult to control their correlation. Nevertheless, to achieve the purpose of this study, we set the options to make the correlation between Amount and CE (−0.12) and the correlation between DI and CE (0.37) as low as possible. The amount of money for the partner and the participant in the options, DI, and CE are listed in Supplementary Table S1.

Following the experiment, one single trial was randomly selected (fMRI, one from 80 trials; TMS, one from 160 trials). The participants received the amount of money in the selected trial. If the participant rejected the option in the selected trial, the participant received nothing.

In the fMRI experiment, the visual stimuli were presented using an LCD projector (WUX5000, Canon Inc.) and a semi-opaque screen placed at the edge of the MR bore. The participants viewed the stimuli via a mirror attached to the head coil. An MRI-compatible response pad (HHSC-1x4-D, Current Designs, PA, USA) was used. In the TMS experiment, a 17-inch laptop PC was used to present the stimuli and record button presses. A response pad used in the TMS experiment (OTR-1x4-D, Current Designs, PA, USA) was similar to that used in the fMRI experiment. PsychoPy software (Peirce et al. 2019) was used to control the stimulus presentation and record the responses in both the fMRI and TMS experiments.

Here, we used a repeated UG where the partner was the same on every trial, whereas previous studies used a one-shot game where participants played UG with a different partner on each trial (Sanfey et al. 2003). If the acceptance/rejection can influence the partner’s choices in subsequent trials, the behavioral motivation for iterated trials is different from that for a one-shot situation. It is noted that the participants were instructed that the partner had chosen the distribution options before the fMRI experiment. Therefore, the participants knew that their acceptance/rejection did not influence the partner’s choices.

### Modeling of behavior

We analyzed the acceptance/rejection of options using a mixed-effects logistic regression. DU in the *t*-th trial of the participants was modeled as follows.

$$ \mathrm{Model}\ 1:U(t)={\beta}_A{x}_A(t)+{\beta}_{DI}{x}_{DI}(t)+{\beta}_{CE}{x}_{CE}(t)+{\beta}_0+\varepsilon . $$

where *x_A_* is the Amount, *x_DI_* is the DI, *x_CE_* is the CE, ${\beta}_A$ is the coefficient for Amount, ${\beta}_{DI}$ is the coefficient for DI, ${\beta}_{CE}$ is the coefficient for CE, ${\beta}_0$ is a constant, and $\varepsilon$ is an error term. The participants were treated as the random effects. Amount, DI, and CE were treated as the fixed effects. The probability of acceptance was estimated as below.

$$ P(t)=1/\left(1+\exp \left(-U(t)\right)\right) $$

Our model (i.e. Model 1) was compared with the following models in Akaike’s information criterion, with lower value considered more appropriate (Akaike 1974).

$$ \mathrm{Model}\ 2:U(t)={\beta}_A{x}_A(t)+{\beta}_{DI}{x}_{DI}(t)+{\beta}_0+\varepsilon $$

$$ \mathrm{Model}\ 3:U(t)={\beta}_A{x}_A(t)+{\beta}_{CE}{x}_{CE}(t)+{\beta}_0+\varepsilon $$

$$ \mathrm{Model}\ 4:U(t)={\beta}_A{x}_A(t)+{\beta}_0+\varepsilon $$

Model 2 is a variant of Fehr-Schmidt model (Fehr and Schmidt 1999) that includes Amount and DI. Model 3 includes Amount and CE. Model 4 is the most parsimonious model that considered only Amount.

We also analyzed the fairness rating scores using a mixed-effects linear regression model similarly to DU. In both analyses for the decision of acceptance/rejection and the fairness rating, the beta coefficients were estimated using *fitglme* function equipped in Statistics and Machine Learning Toolbox of MATLAB software (MathWorks, Inc., MA, USA). The values of Amount, DI, and CE were normalized to Z-scores (mean = 0, standard deviation = 1).

In addition, we prepared three models as below to analyze the acceptance/rejection of options in the fMRI experiment.

$$ \mathrm{Model}\ 5:U(t)={\beta}_{SA}{x}_{SA}(t)+{\beta}_0+\varepsilon $$

$$ \mathrm{Model}\ 6:U(t)={\beta}_0+\varepsilon $$

Model 5 considered the sum of amounts for the participant and partner, *x_SA_*. ${\beta}_{SA}$ is the coefficient for the sum of amounts. Model 6 only included the constant term. We also prepared a model that Amount, DI, and CE were treated as random effects as with participants (Model 7).

### MRI procedures

All MRI data were acquired using a 3-T MRI scanner at Juntendo University Hospital (Siemens Prisma, Erlangen, Germany) with a 32-ch head coil. T1-weighted structural images were obtained using a 3D magnetization-prepared rapid gradient-echo sequence (resolution = 0.8  × 0.8 × 0.8 mm^3^). Functional images were obtained using a multiband gradient-echo echo-planar imaging (EPI) sequence (repetition time = 1.0 s, echo time = 30 ms, flip angle = 59°, in-plane field of view [FOV] = 192 × 192 mm, matrix size = 96 × 96, 72 contiguous slices with no gap, phase encoding direction = posterior–anterior, no parallel acquisition, and multiband factor = 6) (Feinberg et al. 2010; Xu et al. 2013). Single images with the encoding direction of anterior–posterior and posterior–anterior were acquired using a spin-echo EPI sequence with the same FOV and resolution of the gradient-echo EPI sequence. These two images were used for the processing of top-up distortion correction (Andersson et al. 2003). The participants performed the task during four fMRI sessions. Each fMRI session yielded 290 volumes. In total, 1,160 volumes were acquired for each participant.

### Image analyses

The structure images were bias-field-corrected using the AFNI software (Tustison et al. 2010). The functional images were motion-corrected, distortion-corrected using the top-up tool, and spatially normalized into the standard space of Montreal Neurological Institute (MNI) coordinates using the structure image as a reference. We performed general linear model (GLM) analyses following spatial smoothing (full width half maximum = 6 mm) and high-pass filtering (cut-off = 64 s). The presentation of distribution options, the button presses, and the fairness rating were modeled in the design matrix of the GLM. The Amount, DI, and CE were entered into the GLM as parametric modulations for the presentation of the distribution options. Six head motion parameters calculated in the realignment were included as nuisance regressors. We used SPM12 (www.fil.ion.ucl.ac.uk/spm/software/spm12/) to analyze the functional images except for the processing of top-up implemented in FSL (Smith et al. 2004). We turned off the orthogonalization option for the parametric modulations (Mumford et al. 2015).

The cluster forming threshold was set to *P* < 0.001, and the cluster-level statistics values were calculated in consideration of the family-wise error. We also performed small volume correction for the activation for the parametric modulation of Amount and DI, taking advantage of activation results reported in previous studies. Specifically, the small volume for the activation associated with Amount was defined using the term-based meta-analysis available on the Neurosynth website (Yarkoni et al. 2011). The statistical map generated from the term “money” showed two top peaks located in the left and right ventral striatum, and the combination of two 6-mm radius spheres centered at the two top peaks (Supplementary Fig. S1A) was used as the small volume for Amount. In contrast, the small volume for DI was generated from a previous study (Halko et al. 2009), due to the rarity of previous studies of DI, and was defined as a 6-mm radius sphere (Supplementary Fig. S1B) centered at the activation peak.

We carried out psychophysiological interaction (PPI) analyses with the seeds of VS, dAG, and vAG using the gPPI toolbox (McLaren et al. 2012), and with the target of the SMG. The seed and target regions were defined as a 6-mm radius sphere centered at the peak coordinates of the brain activation associated with the parametric modulations. We used MarsBaR toolbox for SPM (Brett et al. 2002) to define regions of interest (ROIs) and identify the beta estimates of functional connectivity in the SMG.

We used DCM 12.5 (Friston et al. 2003; Li et al. 2011) implemented in SPM12 to identify the directionality of connectivity based on the functional connectivity analyses. The design matrix of the DCM analysis included a regressor for the presentation of distribution and six nuisance regressors of head motion. We prepared three DCM models, including bidirectional, forward, and backward connectivity of VS-SMG, dAG-SMG, and vAG-SMG (Fig. 4C), and performed a fixed-effects BMS to determine the best model of the three models (Stephan et al. 2010). In addition, a random-effects BMS, encompassing the differences in the best models of the participants, was also carried out to corroborate the results of the fixed-effects BMS.

### TMS procedures

TMS was administered using a 70-mm figure-of-eight coil (D70 Alpha BI Coil) connected to a Magstim Rapid^2^ (The Magstim Company Ltd, Spring Gardens, Whitland, UK) that is a non-invasive biphasic magnetic stimulator. Sigle pulse stimulations were administered to assess the active motor threshold (AMT) for the right first dorsal interosseous (FDI). Ag/AgCl adhesive electrodes were placed over the muscle belly as an active electrode and the metacarpophalangeal joint of the index finger as a reference electrode. The signals were sent to an amplifier through a band-pass filter from 5 Hz to 3 kHz. The AMT was defined as the lowest intensity that evoked a small response (>100 mV) more than 5/10 trials when the right FDI was contracted approximately 10% of the maximum voluntary contraction (Han et al. 2019; Tamura et al. 2019; Osada et al. 2021).

The stimulation was targeted to the vAG associated with CE using a navigator system (Localite GmbH, Bonn, Germany). A T1-weighted image of each participant was registered to the navigator system to indicate the target position in the participant’s native space. The position and orientation of the coil were calibrated in the native space. The navigation system recorded the position and orientation of the coil during the stimulation. The TMS coil was placed such that the short axis of the coil was approximately perpendicular to the superior temporal sulcus.

We administered a cTBS to the vAG twice (i.e. real and sham stimulations). The cTBS lasted 40 s, during which five bursts of three 50-Hz pulses were delivered every second (Huang et al. 2005). During the sham stimulation, the coil was set perpendicular to the position of the real stimulation to avoid stimulating the brain. The intensity of cTBS was set to 80% of the AMT for each participant. The average stimulation intensity was 35.7% (standard deviation = 2.3) of the maximum output of the stimulator. The task started five minutes after completion of each of the real and sham cTBS. The order of real and sham stimulation was counterbalanced across participants. In each of the first and second halves, participants performed the task over the course of 80 trials. The settings of distribution options were identical to the fMRI experiment (Supplementary Table S1). The participants performed 160 trials in total in the TMS experiment.

## Results

### Behavioral results of fMRI experiments

In each trial of a modified UG in this study (Fig. 1A), two distribution options were presented on the left and right sides of the display. Each option reflected the amount of money for the partner (shown Saki) and for the participant (shown You). The option that the partner chose was colored yellow (actual option), while the other option was colored white (counterfactual option). The participant decided to accept or reject the actual option. Next, the participants rated the fairness of the distribution in consideration with the counterfactual option (1 = absolutely unfair, 7 = absolutely fair). We modeled DU of the participants as the linear combination of Amount (amount of money for the participant), DI (amount of money for the participant minus that for the partner), and CE (amount of money for the participant in the actual option minus that in the counterfactual option) (Fig. 1B), and analyzed the acceptance/rejection of options using a mixed-effects logistic regression.

**Fig. 2:**
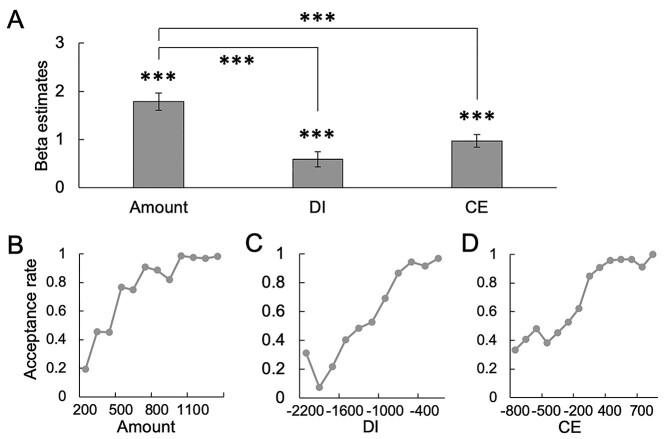
Behavioral results in the fMRI experiment. A. Beta estimates of mixed-effects logistic regression analysis. All beta values were significant. Error bars reflect the standard error. The asterisks indicate the statistical significance (^*^^*^^*^P < 0.001). B. Relation between amount and acceptance rate. C. Relation between DI and acceptance rate. D. Relation between CE and acceptance rate.

Results demonstrated that our model of DU (i.e. Model 1) explained the acceptance/rejection well compared to the other three models (Supplementary Table S2). Our model was also better than the additional three models in terms of AIC (Model 5, 1762.6; Model 6, 1763.7; Model 7, 960.0). Amount, DI, and CE significantly contributed to deciding whether to accept or reject (Fig. 2A). Amount was significantly more influential in the decision than DI (two-tailed Z-test, Z = 5.02, *P* = 0.00000053, uncorrected) and CE (two-tailed Z-test, Z = 3.66, *P* = 0.00025, uncorrected). The participants were more likely to accept distributions as Amount, DI, and CE increased (Fig. 2B–D). The results of the mixed-effects linear regression to assess the fairness rating also showed that our model outperformed the other three models (Supplementary Table S3). Amount, DI, and CE significantly influenced the fairness rating. To examine the behavioral consistency, the mixed-effects logistic regression analysis of our model was applied to the first and second halves of the behavioral data for acceptance/rejection decision and fairness rating. The results showed that the influence of CE and DI was not significantly different between the first and second halves (Supplementary Tables S4 and S5).

### Imaging results of fMRI experiments

We first replicated brain activation in the regions reported in previous studies of the UG (Sanfey et al. 2003; Chang and Sanfey 2009; Harlé et al. 2012; Corradi-Dell’Acqua et al. 2013; Grecucci et al. 2013; Zheng et al. 2015; Shaw et al. 2019). The contrast of “reject vs. accept” was calculated, in analyses separate from the following main analyses, during Options, Response and Feedback periods (Fig. 1A). The contrast during Options period revealed the activation in the right dorsolateral prefrontal cortex (DLPFC) and the contrast during Feedback period revealed brain activity in the insular cortex; however, no prominent activation was observed in the rTPJ in any of these contrasts (Supplementary Fig. S2).

**Table 1:** Brain activation associated with amount, DI, CE, and DU.

Parameter	MNI coordinates of peak			Peak *t* value	Cluster size (voxels)	*P*-FWE-corr
Region	*x*	*y*	*z*			
Amount						
L VS	−14	14	−12	4.09	39	0.041 (SVC)
DI						
R dAG	56	−58	36	4.93	82	0.010 (SVC)
CE						
R vAG	52	−54	20	4.93	110	0.027
DU						
R SMG	48	−32	38	5.19	145	0.017
R pAG	38	−66	36	5.12	335	< 0.001
R Precuneus	18	−64	32	4.83	121	0.037

SVC, small volume correction at voxel-level; L, left; R, right; VS, ventral striatum; dAG, dorsal angular gyrus; vAG, ventral angular gyrus; pAG, posterior angular gyrus; SMG, supramarginal gyrus.

**Fig. 3:**
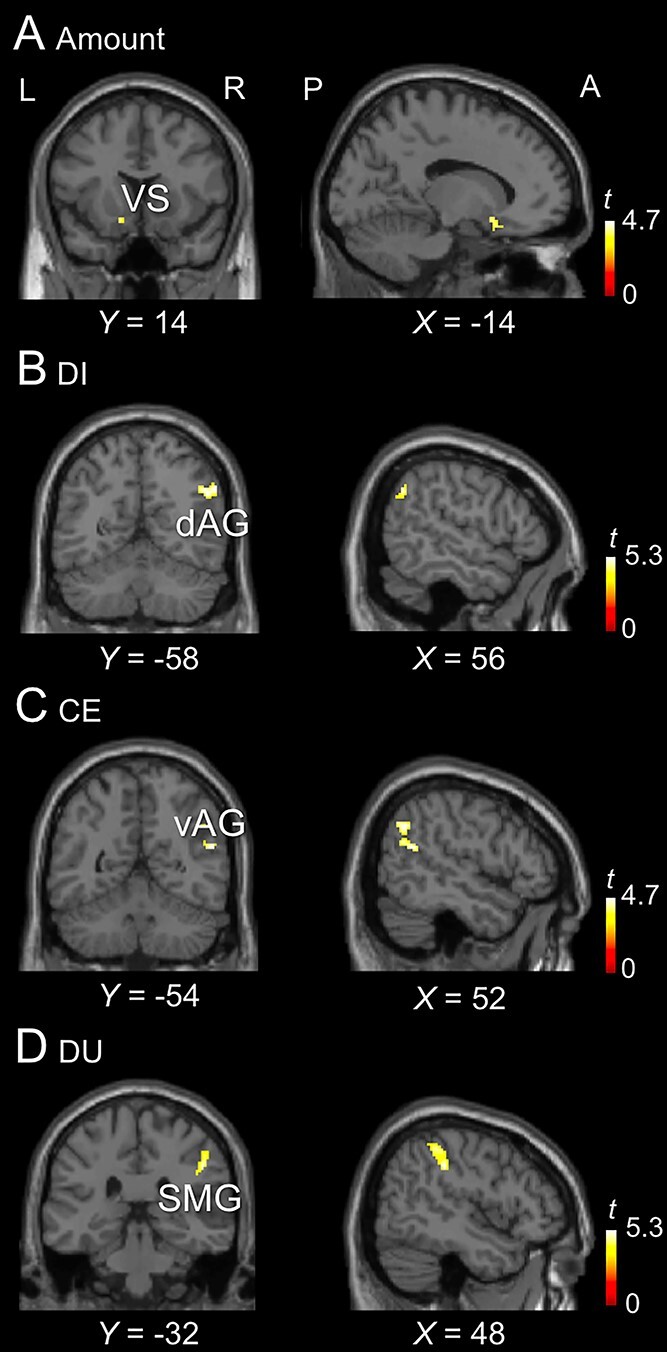
Brain activation. A. Activation associated with amount. B. Activation associated with DI. C. Activation associated with CE. D. Activation associated with DU. VS, ventral striatum; dAG, dorsal angular gyrus; vAG, ventral angular gyrus; SMG, supramarginal gyrus.

Of central interest in this study, we analyzed the brain activation during Options period using the parametric modulation of Amount, DI and CE. The activation for Amount was observed in the ventral striatum (VS) (Fig. 3A), and the activation for DI was observed in the right dorsal angular gyrus (dAG) (Fig. 3B), consistent with previous studies of the UG (Hollmann et al. 2011; Harlé et al. 2012; Haruno et al. 2014; Hétu et al. 2017). Of central interest in this study was the activation associated with CE observed in the right ventral angular gyrus (vAG) (Fig. 3C). DU, the linear sum of Amount, DI and CE, was associated with, primarily, the supramarginal gyrus (SMG) (Fig. 3D). The coordinates, peak values, cluster size, significance level of each activation are listed in Table 1.

**Fig. 4:**
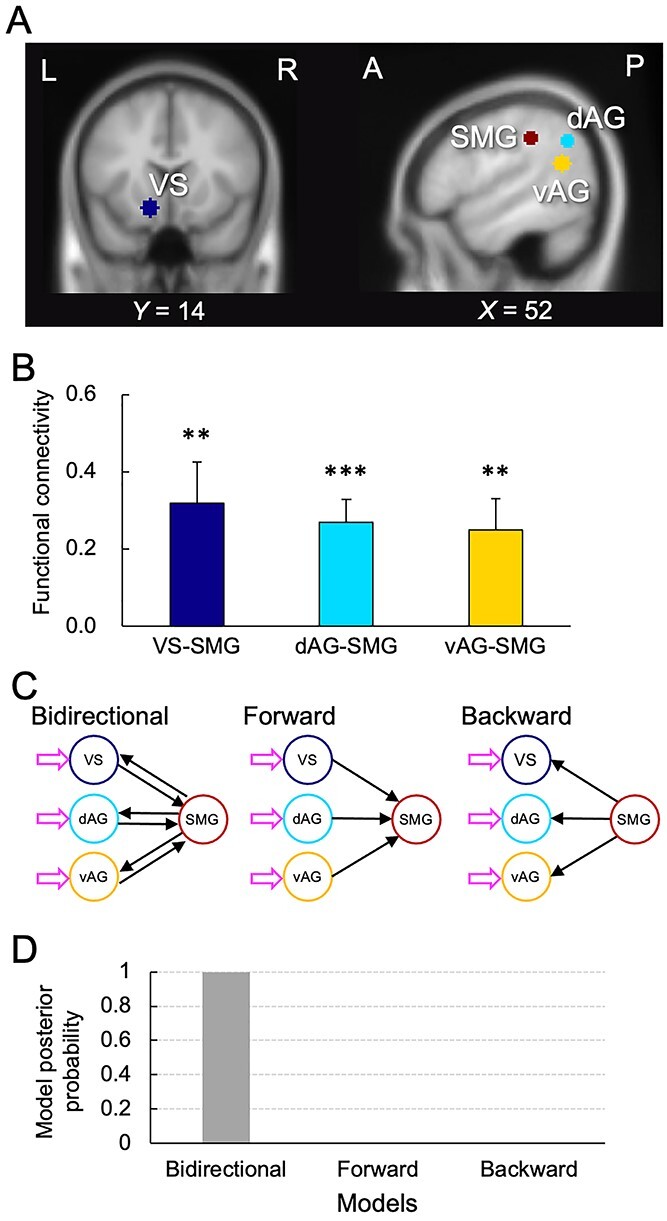
Results of the connectivity analyses. A. ROIs of the component regions (the VS, dAG, and vAG) and the SMG used for connectivity analyses. B. Functional connectivity in VS-SMG, dAG-SMG and vAG-SMG. Error bars indicate standard error. The asterisks indicate the statistical significance (^*^^*^P < 0.01, ^*^^*^^*^P < 0.001). C. Model space. Three models are prepared for DCM. Arrows colored in magenta show the driving inputs. Black arrows indicate endogenous connectivity. D. Result of the Bayesian model comparison. The bidirectional model is the best of three models in terms of model posterior probability.

We analyzed the functional connectivity in our model between each of the component regions (the VS, dAG, and vAG) and the SMG (Fig. 4A). Results revealed that all the functional connectivity (VS-SMG, dAG-SMG, and vAG-SMG) were significant (Fig. 4B). Based on the functional connectivity results, we further carried out a dynamic causal modeling (DCM) analysis to identify the directionality of connectivity. We prepared three DCM models, including bidirectional, forward, and backward connectivity of VS-SMG, dAG-SMG, and vAG-SMG. (Fig. 4C). The results of the fixed-effects Bayesian model comparison (BMS) showed that the bidirectional model was the best of the three models (Fig. 4D). All driving inputs and endogenous connectivity were highly significant in the bidirectional model (Bayesian probability *P* > 0.99, Supplementary Table S6). The random-effects BMS, encompassing the differences in the best models of the participants, also confirmed that the bidirectional model was the best among the three models (Supplementary Fig. S3).

### Behavioral results of TMS experiments

In order to test the causality of the vAG in relation to CE, we administered an inhibitory continuous theta-burst stimulation (cTBS) (Huang et al. 2005) to the vAG in two stimulation conditions: one in the real and the other in sham condition. The response time, acceptance rate and fairness rating were not changed after cTBS (two-tailed paired *t*-test, *t*(19) = −1.31, *P* = 0.21 for the response time; two-tailed paired *t*-test, *t*(19) = 0.40, *P* = 0.69 for the acceptance rate; two-tailed paired *t*-test, *t*(19) = −1.46, *P* = 0.16 for the fairness rating) (Supplementary Fig. S4).

**Fig. 5:**
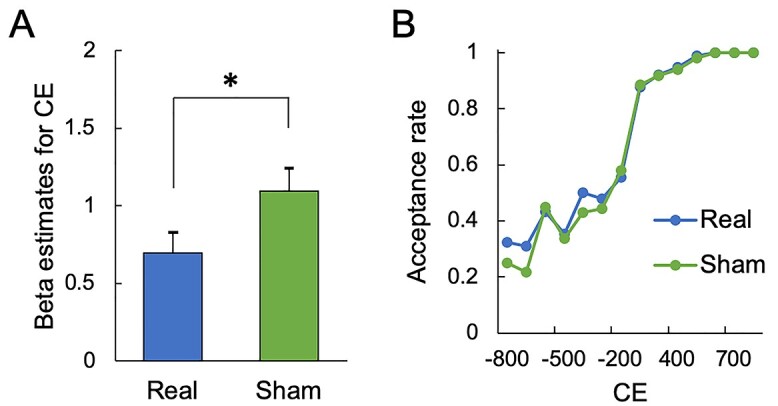
Results of TMS experiment. A. Beta estimates of mixed-effects logistic regression analysis for CE in the real and sham conditions. Error bars reflect standard error. The asterisk indicates the statistical significance (^*^P < 0.05). B. Relationship between CE and acceptance rate in the real and sham conditions.

The mixed-effects logistic regression for the decision of acceptance/rejection showed that participants significantly changed their decision weight on CE in the real stimulation condition compared to the sham stimulation condition (two-tailed Z-test, Z = −2.04, *P* = 0.042) (Fig. 5A). A post hoc sensitivity analysis showed that the effect size of this result (0.69) is above the required effect size (0.66) estimated using G^*^power software (Faul et al. 2007). When participants received less money in the actual option compared to the counterfactual option, in particular, they were more likely to accept the option after the cTBS to the vAG (Fig. 5B, Supplementary Table S7). The point-wise difference between Real vs. Sham in Fig. 5B did not reveal significant results. The results suggest that participants experienced a certain degree of difficulty rejecting it after the cTBS to the vAG.

Similar to the decision of acceptance/rejection, Amount, DI, and CE influenced the fairness rating (Supplementary Table S8). However, the cTBS did not change CE weight in fairness rating (two-tailed Z-test, Z = 0.32, *P* = 0.75). Since the vAG contributed to the rejection decision in terms of CE but not fairness evaluation, the participants may have made acceptance decisions (Fig. 5B) recognizing unfairness following cTBS to the right vAG. The altered accept/reject behavior following repetitive TMS without changing subjective rating has also been reported in a previous study of the UG, although the target region was the dorsolateral prefrontal cortex (Knoch et al. 2006).

## Discussion

This study investigated, using fMRI, the neural basis of CE and how CE was integrated with DI into DU. We further assessed, using TMS, the causal role of the brain region associated with CE. We modeled CE, as well as Amount and DI, in DU. The behavioral results in the fMRI experiment revealed that the participants considered CE, as well as Amount and DI, during the decision of acceptance/rejection. Imaging results demonstrated that the right vAG in the rTPJ was associated with CE and that the three component regions converged in the right SMG. It was further confirmed that the inhibitory stimulation of the vAG reduced CE during the decision when the amount for the participant was less in the actual option compared to the counterfactual option. These suggest that vAG contributes to rejecting an unfair distribution when the partner could have chosen a fairer distribution option.

Previous studies using the UG have mainly highlighted the involvement of the DLPFC and insula during the decision of acceptance/rejection of distribution options (Sanfey et al. 2003; Chang and Sanfey 2009; Harlé et al. 2012; Corradi-Dell’Acqua et al. 2013; Grecucci et al. 2013; Shaw et al. 2019). In contrast, this study modeled the components of Amount, DI, and CE and revealed brain activity in the rTPJ using the parametric modulation analysis. The brain activity related to these components in the rTPJ is thus orthogonal to the brain activity in the contrast of rejection vs. acceptance reported in the previous studies.

The activation related to DU was observed in the SMG using the model-based analysis. The connectivity analysis further revealed the significant reciprocal connectivity between the SMG and each of the component regions (VS for Amount; dAG for DI; vAG for CE). The previous meta-analytic studies suggest that the SMG represents a region for integrating sensory stimuli and contextual information to make behavioral responses (Jakobs et al. 2012; Bzdok et al. 2013). Consistent with our model, the component processes, Amount, DI, and CE, seem to converge in the SMG associated with DU. These suggest that the SMG integrates Amount, DI, and CE in order to decide whether to accept or reject the distribution option.

Previous studies using UG used a one-shot game where participants played with a different partner on each trial (Sanfey et al. 2003). In a repeated game, it is rational to behave fairly and reject unfair distributions because the participants’ decisions can influence the subsequent partner’s choices (Hertz et al. 2017; Hill et al. 2017; Na et al. 2021). Thus, such long-time motivation may be weighted rather than short-term motivation in a repeated game. In this study, we used a repeated version of UG, where the participants understood that the partner had chosen the distribution options in advance, and their acceptance/rejection would not influence the partner’s choices in subsequent trials. Therefore, the long-time motivation might be minimally weighted in this study.

Our fMRI results revealed that the right vAG in the rTPJ was associated with CE. Previous fMRI and TMS studies suggested that the rTPJ plays an important role in a number of social cognition situations, as exemplified by cognitive perspective-taking of others (Saxe and Kanwisher 2003; Saxe and Wexler 2005; Corradi-Dell’Acqua et al. 2008; Van Overwalle 2009; Santiesteban et al. 2017; O’Connell et al. 2018; Martin et al. 2019, 2020; Ogawa and Kameda 2019), decision-making for self and others (Kameda et al. 2016; Ogawa et al. 2018; Konovalov et al. 2021), and moral judgment (Young and Saxe 2009; Young et al. 2010; Koster-Hale et al. 2013; Yoder and Decety 2014; Chakroff et al. 2016; Obeso et al. 2018). In relation to contextual fairness, one of the two main approaches to human economic behavior with respect to fairness (Sandbu 2007), CE relies on the context that the actual option was chosen out of two distribution options presented to the partner. Among the social processing of the rTPJ, the perspective-taking of the partner (Saxe and Kanwisher 2003; Saxe and Wexler 2005; Corradi-Dell’Acqua et al. 2008; Van Overwalle 2009; Santiesteban et al. 2017; O’Connell et al. 2018; Martin et al. 2019, 2020; Ogawa and Kameda 2019), who made a choice from the two distribution options, may be an important element involved in the cognitive processing of CE.

## Supplementary Material

Suppl_R1_clear_bhac252
