# Supplementary material for: Role of right temporoparietal junction for counterfactual evaluation of partner’s decision in ultimatum game
Source: Cereb Cortex. 2022 Jun 19;33(6):2947–57. doi: 10.1093/cercor/bhac252 (PMC10016052; doi:10.1093/cercor/bhac252)
Supplement: Suppl_R1_clear_bhac252 [file suppl_r1_clear_bhac252.pdf]

Supplementary Materials for

Role of right temporoparietal junction for counterfactual evaluation of partner's decision in ultimatum game

Akitoshi Ogawa, Saki Asano, Takahiro Osada, Masaki Tanaka, Reia Tochigi,

Koji Kamagata, Shigeki Aoki, Seiki Konishi

Table S1. Settings of distribution options.

	A		B					
	Partner	Participant	Partner	Participant	DI_A	DI_B	|CE|	Total
1	800	800	1000	600	0	600	200	1600
2	800	800	1100	500	0	400	300	1600
3	800	800	1300	300	0	1000	500	1600
4	1000	600	1300	300	400	1000	300	1600
5	1000	600	1400	200	400	1200	400	1600
6	1100	500	1300	300	600	1000	200	1600
7	1100	500	1400	200	600	1200	300	1600
8	1300	300	1400	200	1000	1200	100	1600
9	1000	1000	1600	400	0	1200	600	2000
10	1000	1000	1400	600	0	800	400	2000
11	1200	800	1400	600	400	800	200	2000
12	1200	800	1600	400	400	1200	400	2000
13	1200	800	1800	200	400	1600	600	2000
14	1400	600	1600	400	800	1200	200	2000
15	1400	600	1800	200	800	1600	400	2000
16	1600	400	1800	200	1200	1600	200	2000
17	1100	1100	1300	900	0	400	200	2200
18	1100	1100	1500	700	0	800	400	2200
19	1100	1100	1800	400	0	1400	700	2200
20	1300	900	1800	400	400	1400	500	2200
21	1300	900	2000	200	400	1800	700	2200
22	1500	700	1800	400	800	1400	300	2200
23	1500	700	2000	200	800	1800	500	2200
24	1800	400	2000	200	1400	1800	200	2200
25	1200	1200	1400	1000	0	400	200	2400
26	1200	1200	1700	700	0	1000	500	2400
27	1200	1200	1900	500	0	1400	700	2400
28	1400	1000	1700	700	400	1000	300	2400
29	1400	1000	1900	500	400	1400	500	2400
30	1700	700	1900	500	1000	1400	200	2400
31	1700	700	2200	200	1000	2000	500	2400
32	1900	500	2200	200	1400	2000	300	2400
33	1400	1400	1700	1100	0	600	300	2800
34	1400	1400	2000	800	0	1200	600	2800
35	1400	1400	2200	600	0	1400	800	2800
36	1700	1100	2200	600	600	1600	500	2800
37	1700	1100	2500	300	600	2200	800	2800
38	2000	800	2200	600	1200	1600	200	2800
39	2000	800	2500	300	1200	2200	500	2800
40	2200	600	2500	300	1600	2200	300	2800

Table S2. Results of parameter estimation (Amount, DI, and CE (mean ± SEM)) and model comparison (AIC) for decisions of acceptance/rejection in the fMRI experiment.

	AIC	Amount	DI	CE
**Model1**	**936.0**	1.78 ± 0.18<br>P < 0.001	0.59 ± 0.16<br>P < 0.001	0.97 ± 0.13<br>P < 0.001
Model2	993.5	1.85 ± 0.17<br>P < 0.001	1.08 ± 0.14<br>P < 0.001	-
Model3	948.2	2.12 ± 0.16<br>P < 0.001	-	1.20 ± 0.12<br>P < 0.001
Model4	1057.6	2.61 ± 0.14<br>P < 0.001	-	-

Table S3. Results of parameter estimation (Amount, DI, and CE (mean ± SEM)) and model comparison (AIC) for fairness rating in the fMRI experiment.

	AIC	Amount	DI	CE
**Model1**	**5154.6**	0.60 ± 0.05<br>P < 0.001	0.59 ± 0.05<br>P < 0.001	0.38 ± 0.04<br>P < 0.001
Model2	5226.7	0.68 ± 0.05<br>P < 0.001	0.79 ± 0.05<br>P < 0.001	-
Model3	5266.0	0.93 ± 0.04<br>P < 0.001	-	0.57± 0.04<br>P < 0.001
Model4	5452.4	1.30 ± 0.03<br>P < 0.001	-	-

Table S4. Results of parameter estimation (Amount, DI, and CE (mean ± SEM)) of Model1 for decisions of acceptance/rejection in the first and second halves of the fMRI experiment.

	Amount	DI	CE
First half	1.39 ± 0.23<br>P < 0.001	0.84 ± 0.21<br>P < 0.001	0.84 ± 0.17<br>P < 0.001
Second half	2.83 ± 0.34<br>P < 0.001	0.37 ± 0.27<br>P = 0.16	1.33 ± 0.23<br>P < 0.001
Difference	Z = 3.51<br>P < 0.001	Z = -1.35<br>P = 0.18	Z = 1.67<br>P = 0.095

Table S5. Results of parameter estimation (Amount, DI, and CE (mean ± SEM)) of Model1 for fairness rating in the first and second halves of the fMRI experiment.

	Amount	DI	CE
First half	0.58 ± 0.07<br>P < 0.001	0.63 ± 0.08<br>P < 0.001	0.33 ± 0.06<br>P < 0.001
Second half	0.61 ± 0.07<br>P < 0.001	0.54 ± 0.08<br>P < 0.001	0.43 ± 0.06<br>P < 0.001
Difference	Z = -0.39<br>P = 0.70	Z = 0.12<br>P = 0.90	Z = -0.12<br>P = 0.90

Table S6. Result of Bayesian parameter averaging.

	Connectivity	Strength	Probability
Endogenous connectivity	VS to SMG	0.014	1.00
	dAG to SMG	0.017	1.00
	vAG to SMG	0.017	1.00
	SMG to VS	0.014	1.00
	SMG to dAG	0.019	1.00
	SMG to vAG	0.020	1.00
	VS to VS	-0.11	1.00
	dAG to dAG	-0.090	1.00
	vAG to vAG	-0.097	1.00
	SMG to SMG	-0.089	1.00
Driving input of distribution options	VS	-0.0044	1.00
	dAG	-0.0032	1.00
	vAG	-0.0038	1.00

Table S7. Results of parameter estimation (Amount, DI, and CE (mean ± SEM)) and model comparison (AIC) for decisions of acceptance/rejection in the TMS experiment.

	AIC	Amount	DI	CE
Real stimulation				
**Model1**	**811.7**	3.02 ± 0.23<br>P < 0.001	0.90 ± 0.17<br>P < 0.001	0.70 ± 0.13<br>P < 0.001
Model2	838.0	3.11 ± 0.22<br>P < 0.001	1.20 ± 0.16<br>P < 0.001	-
Model3	839.0	3.43 ± 0.22<br>P < 0.001	-	0.97 ± 0.13<br>P < 0.001
Model4	901.4	3.81 ± 0.21<br>P < 0.001	-	-
Sham stimulation				
**Model1**	**814.6**	3.03 ± 0.23<br>P < 0.001	0.54 ± 0.17<br>P = 0.007	1.10 ± 0.14<br>P < 0.001
Model2	879.5	3.05 ± 0.21<br>P < 0.001	1.01 ± 0.15<br>P < 0.001	-
Model3	823.2	3.32 ± 0.21<br>P < 0.001	-	1.27 ± 0.13<br>P < 0.001
Model4	901.4	3.69 ± 0.20<br>P < 0.001	-	-

Table S8. Results of parameter estimation (Amount, DI, and CE (mean ± SEM)) and model comparison (AIC) for fairness rating in the TMS experiment.

	AIC	Amount	DI	CE
Real stimulation				
**Model1**	**4488.2**	0.89 ± 0.04<br>P < 0.001	0.72 ± 0.04<br>P < 0.001	0.29 ± 0.03<br>P < 0.001
Model2	4552.4	0.97 ± 0.04<br>P < 0.001	0.85 ± 0.04<br>P < 0.001	-
Model3	4736.0	1.32 ± 0.04<br>P < 0.001	-	0.50 ± 0.04<br>P < 0.001
Model4	4923.4	1.66 ± 0.03<br>P < 0.001	-	-
Sham stimulation				
**Model1**	**4254.3**	0.89 ± 0.04<br>P < 0.001	0.71 ± 0.05<br>P < 0.001	0.27 ± 0.04<br>P < 0.001
Model2	4677.2	0.97 ± 0.04<br>P < 0.001	0.84 ± 0.04<br>P < 0.001	-
Model3	4846.6	1.32 ± 0.04<br>P < 0.001	-	0.48 ± 0.04<br>P < 0.001
Model4	5010.2	1.65 ± 0.03<br>P < 0.001	-	-

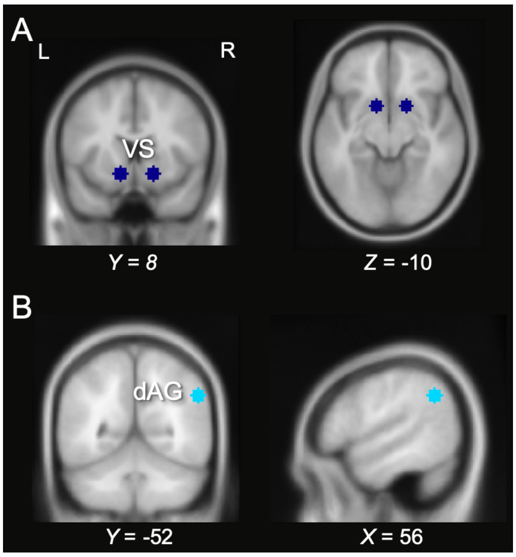

Fig. S1. Small volumes for the parametric modulations of Amount and DI. **A.** Small volume for Amount defined using the term-based meta-analysis available in the website of Neurosynth. The statistical map generated from the term “money” shows two top peaks located in the ventral striatum. The small volume represents the combination of two 6-mm radius spheres centered at the two top peaks. **B.** Small volume for DI defined as a 6-mm radius sphere. The center of the sphere was the peak reported in a previous study (Halko et al., 2009).

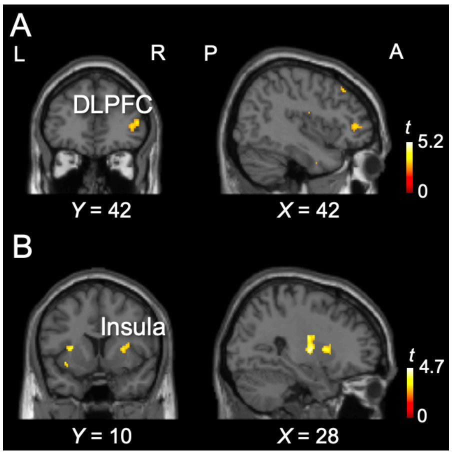

Fig. S2. Brain activation of the contrast “Reject > Accept.” The cluster forming threshold was liberally set to P < 0.01, and the cluster size threshold was 20 voxels. **A.** The activation during Offer period in the right dorsolateral prefrontal cortex (DLPFC). **B.** The activation during Feedback period in the insula.

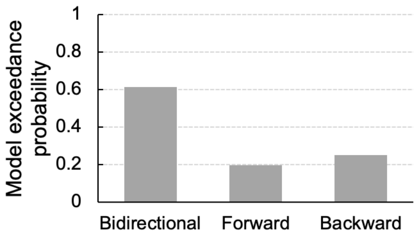

Fig. S3. Result of the random-effects Bayesian model selection. The bidirectional model is the best of three models as the result of the fixed-effects Bayesian model selection.

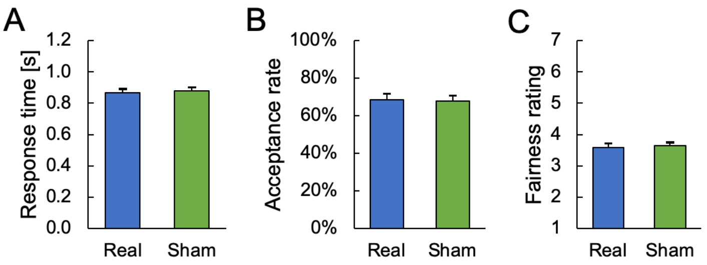

Fig. S4. Behavioral results in the TMS experiment. There was no significant difference between the real and sham conditions in response time (**A**), accept rate (**B**), and fairness rating. (**C**). Error bars reflect the standard error.
